# Supplementary material for: Structure of the human NK cell NKR-P1:LLT1 receptor:ligand complex reveals clustering in the immune synapse
Source: Nat Commun. 2022 Aug 26;13:5022. doi: 10.1038/s41467-022-32577-6 (PMC9418145; doi:10.1038/s41467-022-32577-6)
Supplement: Supplementary file 1 — Supplementary Information [file 41467_2022_32577_MOESM1_ESM.pdf]

Supplementary Information

Structure of the human NK cell NKR-P1:LLT1 receptor:ligand complex reveals clustering in the immune synapse

Jan Bláha[1,5], Tereza Skálová[2], Barbora Kalousková[1,6], Ondřej Skořepa[1], Denis Cmunt[1,7], Valéria Grobárová[3], Samuel Pazicky[1,8], Edita Poláchová[1], Celeste Abreu[1], Jan Stránský[2], Tomáš Koval'[2], Jarmila Dušková[2], Yuguang Zhao[4], Karl Harlos[4], Jindřich Hašek[2], Jan Dohnálek[2], Ondřej Vaněk[1,*]

[1] Department of Biochemistry, Faculty of Science, Charles University, Hlavova 2030, 12840 Prague, Czech Republic

[2] Institute of Biotechnology, The Czech Academy of Sciences, BIOCEV Centre, Průmyslová 595, 25250 Vestec, Czech Republic

[3] Department of Cell Biology, Faculty of Science, Charles University, Viničná 7, 12840 Prague, Czech Republic

[4] Division of Structural Biology, Wellcome Centre for Human Genetics, University of Oxford, Roosevelt Drive, OX3 7BN Oxford, United Kingdom

[5] Present address: EMBL, Hamburg Unit c/o DESY, Notkestrasse 85, 22607 Hamburg, Germany

[6] Present address: Institute of Applied Physics - Biophysics group, TU Wien, Getreidemarkt 9, 1060 Vienna, Austria

[7] Present address: Department of Oncology, Ludwig Institute for Cancer Research, University of Lausanne, Chemin des Boveresses 155, 1066 Epalinges, Switzerland

[8] Present address: School of Biological Sciences, Nanyang Technological University, Nanyang Drive 60, 637551 Singapore

* Correspondence and requests for materials should be addressed to O.V. (email: ondrej.vanek@natur.cuni.cz)

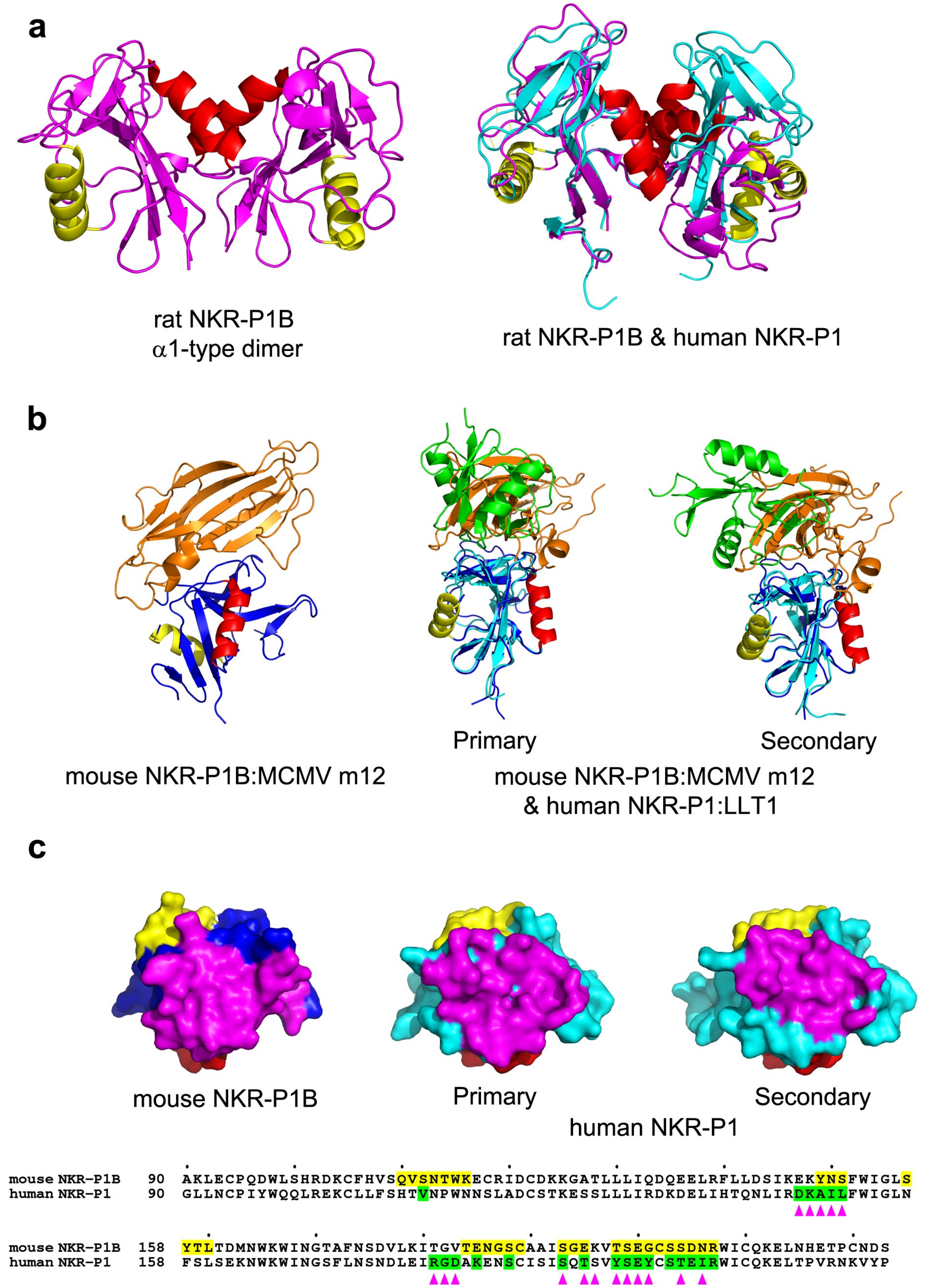

Supplementary Fig. 1 Comparison of rat, murine and human NKR-P1 structures. **a** Comparison between covalent disulfide dimer of rat NKR-P1B (PDB ID 5J2S, magenta) and non-covalent dimer of human NKR-P1 (PDB ID 5MGR, cyan); helices α1 and α2 are shown in red and yellow, respectively. Structural alignment of rat NKR-P1B and human NKR-P1 homodimers, prepared by aligning only one monomer from each dimer, is shown on the right-hand side. **b** Comparison between the structure of mouse NKR-P1B in complex with MCMV immunoevasin m12 (PDB ID 5TZN, blue and orange, respectively) and structure of human NKR-P1 in complex with LLT1 (PDB ID 5MGT, cyan and green, respectively) in primary and secondary interaction modes; helices α1 and α2 are shown in red and yellow, respectively. Structural alignment of the mouse NKR-P1B and human NKR-P1 monomers was used to compare their ligand-bound complexes, as shown on the right-hand side. **c** Interacting residues of NKR-P1 molecules shown in (b) are mapped on their respective surfaces in magenta while other colors follow the coding in (b); all molecules are oriented identically. The identical residues are also highlighted in the sequence alignment below, in the yellow background for mouse NKR-P1B, and with the green background and purple triangles for the primary and secondary interaction mode of human NKR-P1, respectively.

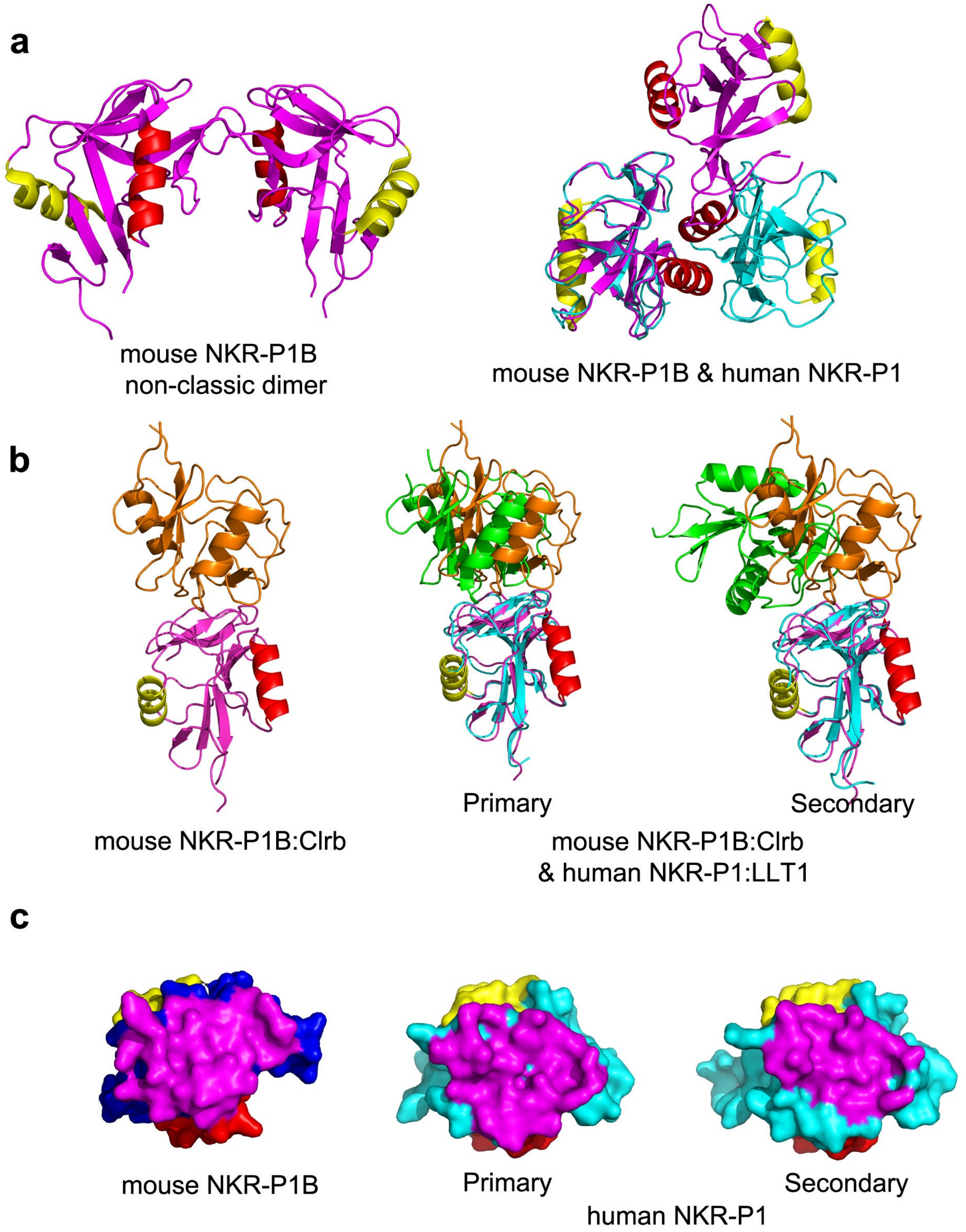


```
mouse NKR-P1B    90  AKLECPQDWLSHRDKCFHVSQVSNTWKECRIDCDKKGATLLLIQDQEELRFLLDSIKEKYNSFWIGLS
human NKR-P1     90  GLLNCPIYWQQLREKCLLFSHTVNPWNNSLADCSTKESSLLLIRDKDELIHTQNLIRDKAILFWIGLN

mouse NKR-P1B   158  YTLTDMNWKWINGTAFNSDVLKITGVTENGSCAAISGEKVTSEGCSSDNRWICQKELNHETPCNDS
human NKR-P1    158  FSLSEKNWKWINGSFLNSNDLEIRGDAKENSCISISQTSVYSEYCSTEIRWICQKELTPVRNKVYP
```

Supplementary Fig. 2 Comparison of murine and human NKR-P1 complexes. **a** Comparison between the non-classical dimer of mouse NKR-P1B (PDB ID 6E7D, magenta) and the non-covalent dimer of human NKR-P1 (PDB ID 5MGR, cyan); helices α1 and α2 are shown in red and yellow, respectively. Structural alignment of mouse NKR-P1B and human NKR-P1 homodimers, prepared by aligning only one monomer from each dimer, is shown on the right-hand side. **b** Comparison between the structure of mouse NKR-P1B in complex with Clrb (PDB ID 6E7D, magenta and orange, respectively) and structure of human NKR-P1 in complex with LLT1 (PDB ID 5MGT, cyan and green, respectively) in primary and secondary interaction modes; helices α1 and α2 are shown in red and yellow, respectively. Structural alignment of the mouse NKR-P1B and human NKR-P1 monomers was used to compare their ligand-bound complexes, as shown on the right-hand side. **c** Interacting residues of NKR-P1 molecules shown in (b) are mapped on their respective surfaces in magenta while other colors follow the coding in (b); all molecules are oriented identically. The identical residues are also highlighted in the sequence alignment below, in the yellow background for mouse NKR-P1B, and with the green background and purple triangles for the primary and secondary interaction mode of human NKR-P1, respectively.

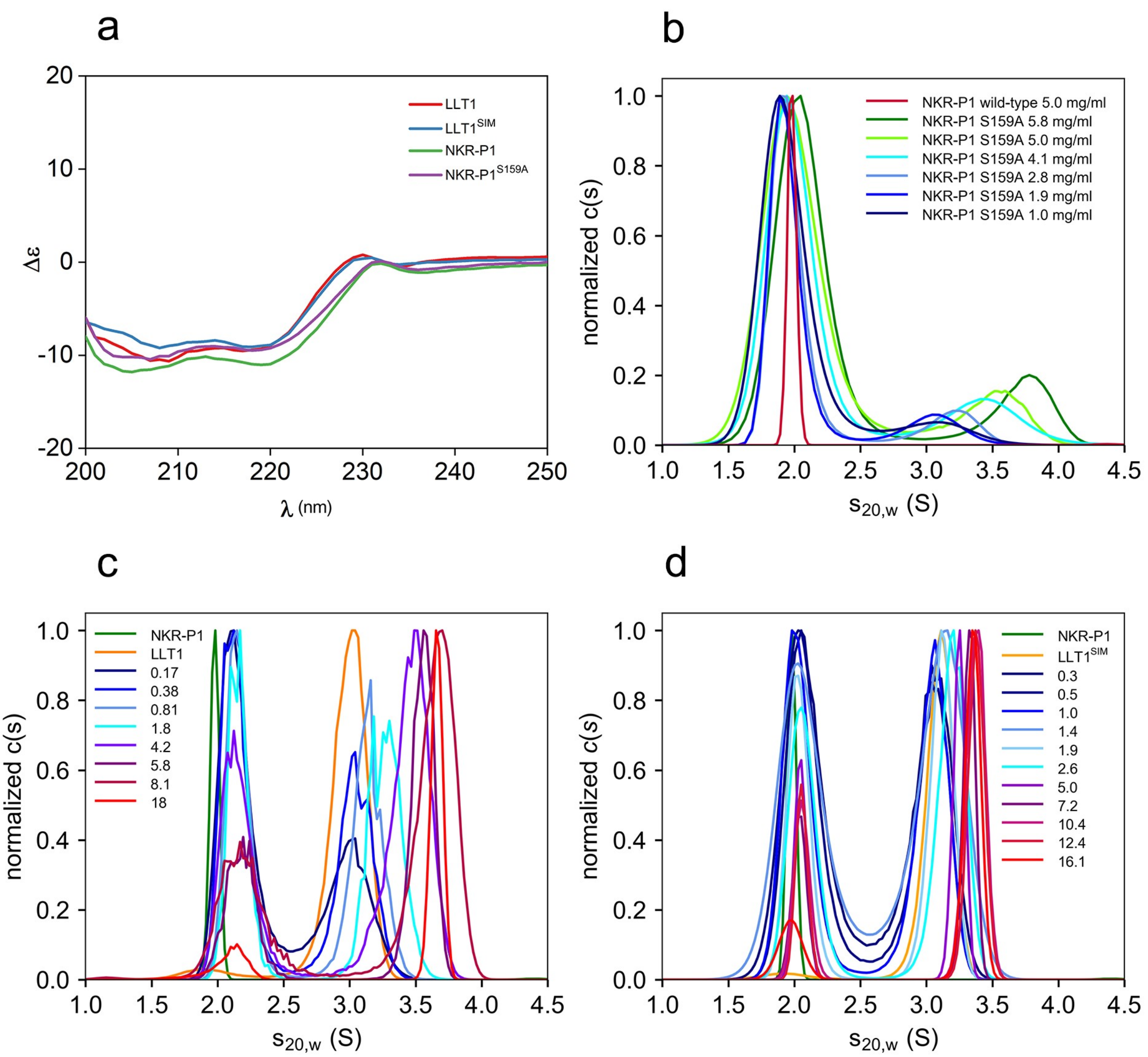

Supplementary Fig. 3 NKR-P1 dimerization and NKR-P1:LLT1 complex formation observed in solution. **a** CD spectra for wild-type LLT1 and NKR-P1 compared to the spectra of LLT1^{SIM} and NKR-P1 S159A mutants used in sedimentation velocity analytical ultracentrifugation experiments. **b** Normalized continuous c(s) distributions of sedimentation coefficient transformed to standard conditions ($s_{20,w}$) for wild-type NKR-P1 (red) and increasing concentrations of NKR-P1 S159A mutant (dark blue to green). The shift of the distributions to higher S values corresponds to NKR-P1 oligomerization. **c** Normalized continuous c(s) distributions of sedimentation coefficient transformed to standard conditions ($s_{20,w}$) for free NKR-P1 (green, 5 mg/ml), free LLT1 (orange, 3.5 mg/ml) and their equimolar mixtures at increasing concentrations (blue to red, total concentration in mg/ml). **d** Normalized continuous c(s) distributions of sedimentation coefficient transformed to standard conditions ($s_{20,w}$) for free NKR-P1 (green, 5 mg/ml), free LLT1^{SIM} (orange, 0.7 mg/ml) and their equimolar mixtures at increasing concentrations (blue to red, total concentration in mg/ml). The shift of the distributions to higher S values in (c) or (d) corresponds to NKR-P1:LLT1 or NKR-P1:LLT1^{SIM} complex formation with fast kinetics, respectively. Source data are provided as a Source Data file.

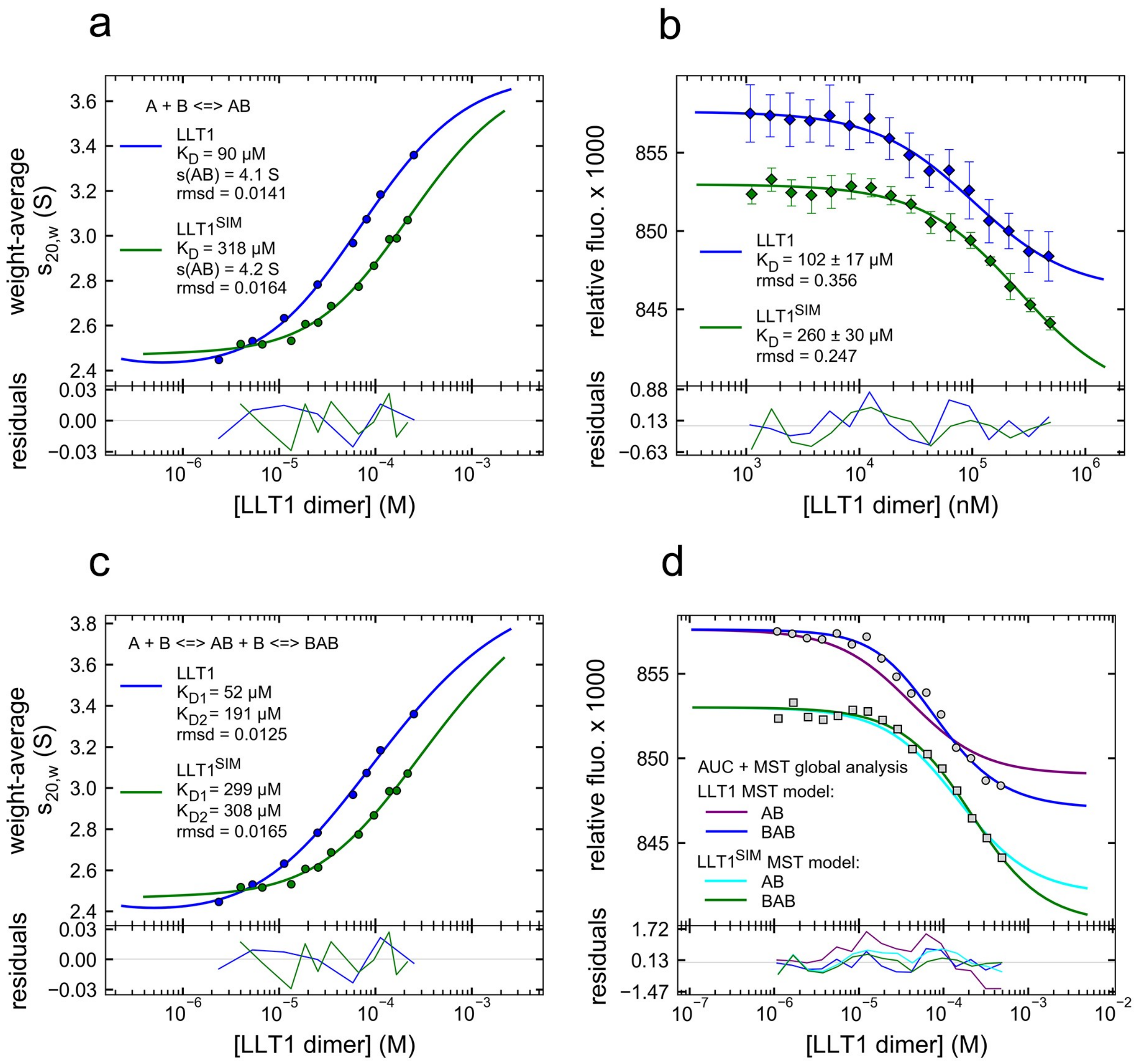

Supplementary Fig. 4 NKR-P1:LLT1 interaction analyzed by analytical ultracentrifugation and microscale thermophoresis. **a,c** Binding isotherms constructed by integrating the distributions shown in Supplementary Fig. 3c and 3d over the whole range and plotting the resulting $s_{20,w}$ values against the LLT1 dimer concentration were best-fit with A+B⇔AB model where A is the LLT1 dimer and B is the NKR-P1 monomer in (a) or with A+B⇔AB+B⇔BAB model in (c). **b** Microscale thermophoresis data from three independent measurements were averaged and best-fit with A+B⇔AB model where A is the LLT1 dimer and B is the NKR-P1 monomer. Data points represent the means of the three experiments, and the whiskers represent ±SD. **d** Global analysis of sedimentation velocity binding isotherms (c) and microscale thermophoresis data (b). Sedimentation parameters were fixed to the previously best-fit values, and the fluorescence isotherms were fit with either the AB or the BAB model. While for the LLT1^{SIM} the AB model describes the data almost equally well as the BAB model, for wild-type LLT1, the AB model shows a poor fit. The LLT1^{SIM} data in (b) and (d) were purposely offset 0.004 relative fluorescence units for clarity. Source data are provided as a Source Data file.

SAXS frame 355-367

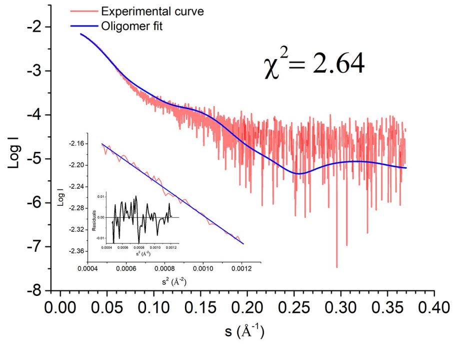

LLT1:NKR-P1 oligomer	*Component percentage*
NP_LL_PP_LL_PN	0.352
**LL_PS_LL_PS_LL_PN**	**0.648**

SAXS frame 368-378

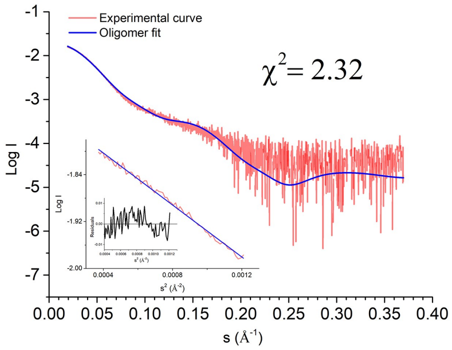

LLT1:NKR-P1 oligomer	*Component percentage*
**NP_LL_PP_LL_PN**	**0.813**
LL_PS_LL_PS_LL_PN	0.187

SAXS frame 379-388

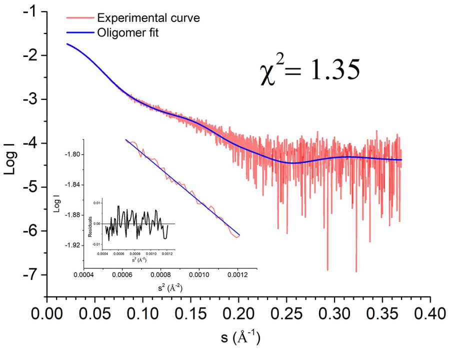

LLT1:NKR-P1 oligomer	*Component percentage*
**S_LL_PS_LL_PN**	**0.841**
NP_LL_PP_LL_PN	0.071
LL_PS_LL_PS_LL_PN	0.088

SAXS frame 389-399

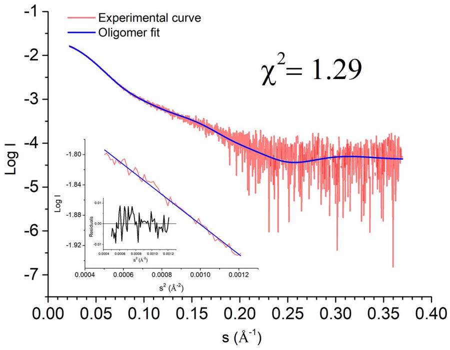

LLT1:NKR-P1 oligomer	*Component percentage*
S_LL_P	0.042
LL_PS_LL	0.234
NS_LL_PN	0.039
**S_LL_PS_LL_PN**	**0.684**

SAXS frame 431-440

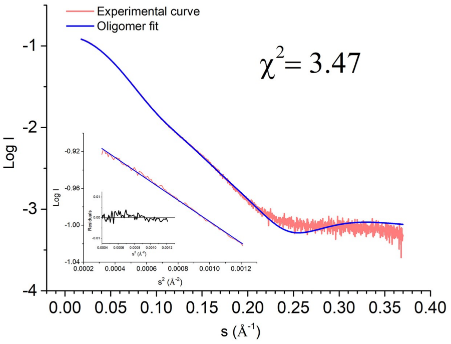

LLT1:NKR-P1 oligomer	*Component percentage*
LL	0.094
NN	0.011
**NS_LL**	**0.479**
S_LL_P	0.09
NP_LL	0.326

SAXS frame 481-491

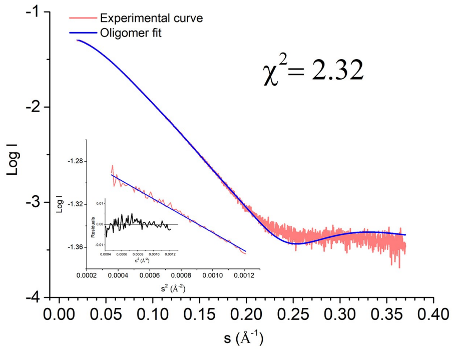

LLT1:NKR-P1 oligomer	*Component percentage*
N	0.215
LL	0.298
NN	0.176
**NS_LL**	**0.31**

Supplementary Fig. 5 OLIGOMER analysis of selected intervals of SEC-SAXS data for NKR-P1:LLT1 complexes. SAXS scattering curves in logarithmic scale (red) with Guinier plots in the lower-left corners and the best-fit scattering curves calculated using OLIGOMER (blue), quality of the OLIGOMER fit is given as a χ^2 value, and the list of the superposed structures and their percentage abundance fitted to calculate the OLIGOMER scattering curve is shown below. L denotes the LLT1 monomer, N the NKR-P1 monomer, and P and S the NKR-P1 monomer engaged in the primary or secondary interaction mode with neighboring LLT1, respectively.

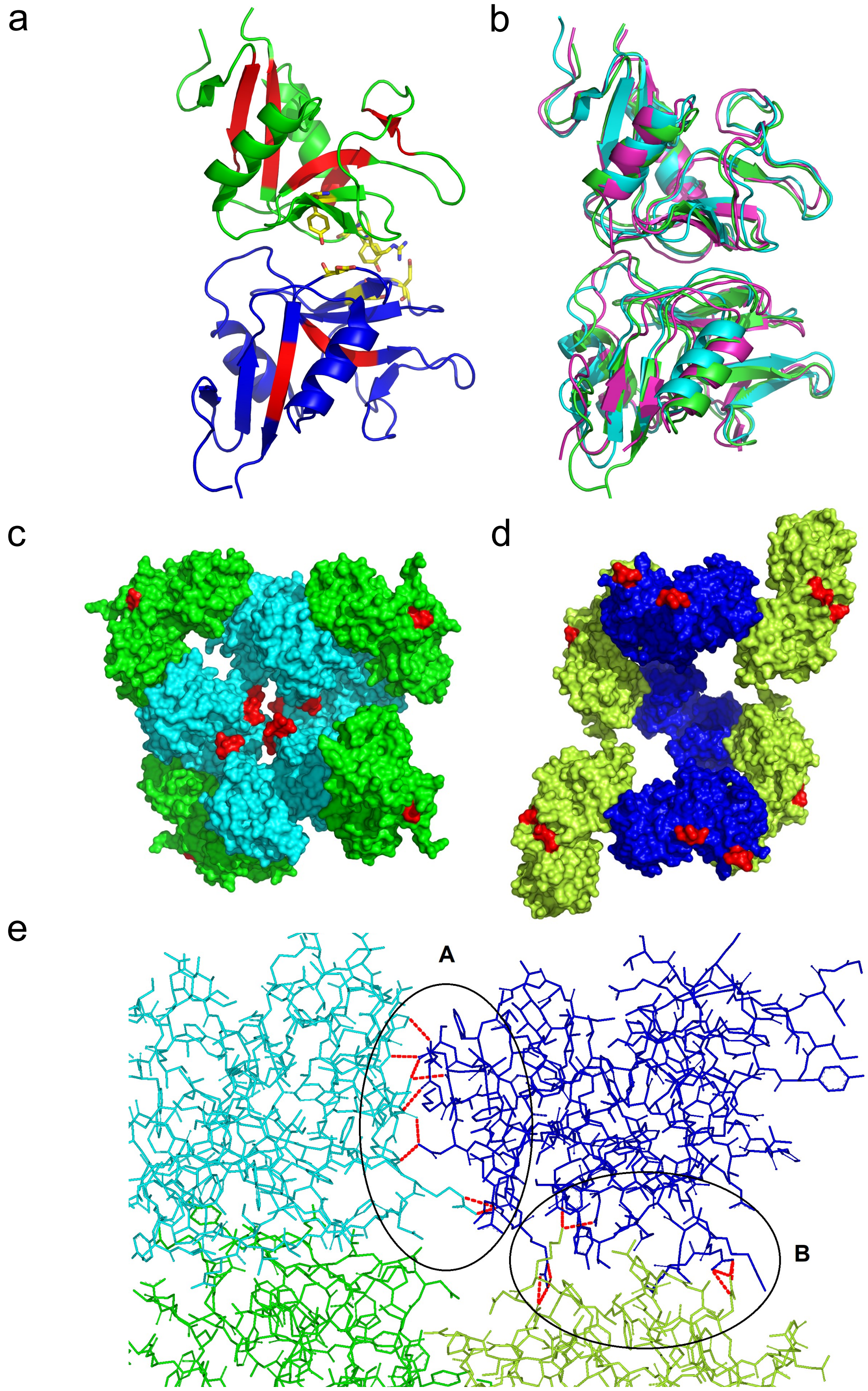

Supplementary Fig. 6 Anatomy of NK cell CTL receptor:ligand interactions. Theoretic *in silico* models of the NKR-P1:LLT1 complex oligomeric assemblies, based on the propagation of only a single interaction mode, are biologically improbable. NKR-P1 accessory interaction interface stabilizes the NKR-P1:LLT1 complex in primary and secondary binding modes. **a** The primary mode of interaction between human NKR-P1 (blue) and LLT1 (green). The residues colored red show conserved sequence fragments at least three residues long corresponding structurally in the superposition of the three receptor:ligand interaction pairs (primary mode of human NKR-P1:LLT1, murine NKR-P1B:Clrb, and human NKp65:KACL). The residues partially conserved at their interaction interface (see Suppl. Table 2) are shown as sticks for the case of NKR-P1:LLT1. **b** Overlapped interaction pairs of the primary mode of human NKR-P1:LLT1 (green), murine NKR-P1B:Clrb (cyan), and human NKp65:KACL (magenta). **c** Theoretic oligomer based on the primary interaction mode only. LLT1 is in green, NKR-P1 in cyan. **d** Theoretic oligomer based on the secondary interaction mode only. LLT1 is in lemon, NKR-P1 is in blue. The models were created by applying symmetry operations to parts of the NKR-P1:LLT1 complex crystal structure to artificially keep and propagate only the primary or the secondary binding mode. The first three N-terminal residues of the proteins are highlighted in red to highlight their orientation. In both (c) and (d), positions of the N-termini are incompatible with the physiological topology of the complex, i.e., the NKR-P1 receptor on the NK cell membrane and LLT1 ligand on the surface of another cell. **e** A detail of the NKR-P1:LLT1 complex structure showing NKR-P1 and LLT1 interacting in the primary binding mode (cyan and green, respectively) and the secondary binding mode (blue and lemon, respectively). The accessory interaction interface (A) between the two neighboring NKR-P1 molecules ligated in the primary and secondary mode (cyan and blue, respectively) has a similar size and number of hydrogen bonds as the secondary interaction mode of the complex (B), thus stabilizing this complex's arrangement. This type of NKR-P1:NKR-P1 contact is not present in the theoretical models depicted in panels (c) and (d) above.

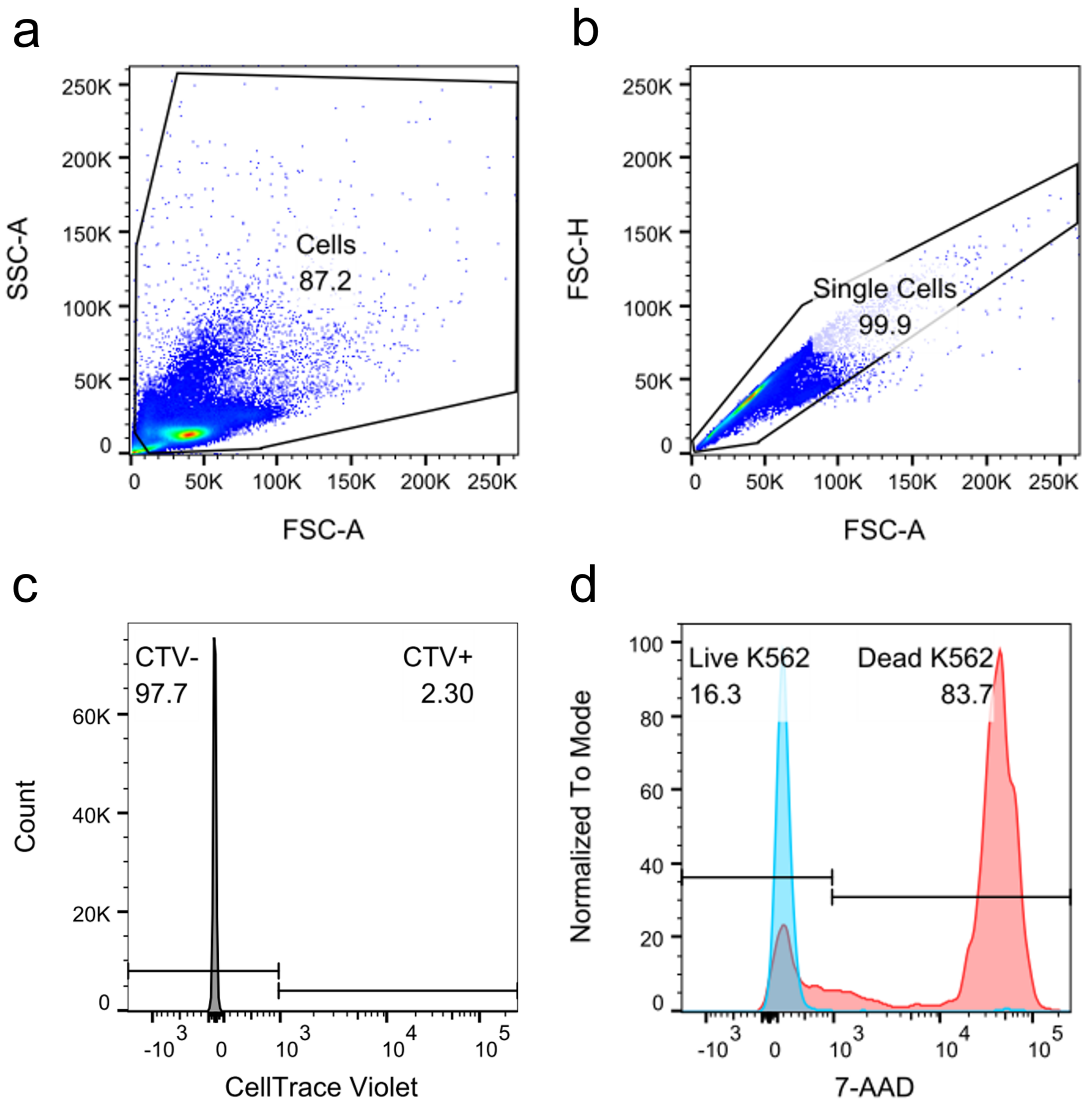

Supplementary Fig. 7 Gating strategy for flow cytometry analysis evaluating LLT1 and LLT1^{SIM} inhibition of NK cell-mediated killing. **a** The population of cells identified from all measured events. **b** To exclude doublets or clusters, the single-cell populations were identified from all cells; there are two cell populations in this gate differing in size (K562 and NK cells). **c** A population of K562 target cells pre-stained with CellTrace Violet (CTV) proliferation reagent was identified from all single cells. **d** Finally, CTV^{+} K562 cells were gated as 7-AAD positive, dead cells and 7-AAD negative, living cells. The control (K562 cells only) is shown in blue, and an exemplary histogram from the cytotoxicity assay (specifically, K562 cells incubated with NK cells and 250 µM LLT1^{SIM}) is shown in red. The resultant percentages of living 7-AAD^{-} CTV^{+} K562 cells were used to construct Fig. 7 in the main text. The same gating was applied to all analyzed samples. FACS data were acquired with BD LSR II flow cytometer and BD Diva software; data were evaluated with FlowJo software.

Supplementary Table 1. Hydrogen bonds in the dimerization interface of NKR-P1, in the primary and secondary binding modes of the NKR-P1:LLT1 complex, and within the mutual contact of NKR-P1 bound in primary and secondary modes. Water-mediated protein-protein hydrogen bonds are not shown.

NKR-P1 – chain A	NKR-P1 – chain B	Hydrogen bonds	Distance [Å]
helix α1-centered dimerization (NKR-P1_glyco: chain A, NKR-P1_glyco: chain B)			
Ser127	Ser123	A/Ser127 O:B/Ser123 Oγ	2.8
Ile168	Thr124	A/Ile168 O:B/Thr124 Oγ1	3.1
Glu213	Glu126	A/Glu213 N:B/Glu126 Oε2	2.8
Ser123	Ser127	A/Ser123 Oγ:B/Ser127 O	2.7
Thr124	Ile168	A/Thr124 Oγ1:B/Ile168 O	3.1
Glu126	Glu213	A/Glu126 Oε2:B/Glu213 N	2.8
Asn157	NAG501	A/Asn157 Nδ2:B/NAG501 O7	3.0
Ser159	NAG501	A/Ser159 Oγ:B/NAG501 O3	3.3
NAG501	Asn157	A/NAG501 O7:B/Asn157 Nδ2	2.9
NAG501	Ser159	A/NAG501 O4:B/Ser159 Oγ	3.3
NAG501	Ser161	A/NAG501 O4:W615/W632:B/Ser161 Oγ	4.8
**LLT1**	**NKR-P1**	**Hydrogen bonds**	**Distance [Å]**
Primary mode (LLT1: chain B, NKR-P1: chain D)			
Ser129	Lys148	Ser129 Oγ:Lys148 O	2.6
Asp130	Ala149	Asp130 N:Ala149 O	3.0
Glu162	Tyr201	Glu162 Oε1:Tyr201 OH	2.5
Arg175	Asp183	Arg175 Nη1:Asp183 Oδ2	2.9
Arg175	Asp183	Arg175 Nη2:Asp183 Oδ2	3.2
Arg175	Glu200	Arg175 N:Glu200 Oε2	3.0
Tyr177	Asp183	Tyr177 OH:Asp183 N	3.2
Tyr177	Ser199	Tyr177 OH:Ser199 O	2.6
Glu179	Arg181	Glu179 Oε1:Arg181 Nη1	3.1
Glu179	Arg181	Glu179 Oε1:Arg181 Nη2	3.0
Glu179	Tyr198	Glu179 N:Tyr198 OH	3.2
Secondary mode (LLT1: chain A, NKR-P1: chain C)			
Asn120	Arg181	Asn120 Oδ1:Arg181 Nη1	3.0
Asn120	Arg181	Asn120 Oδ1:Arg181 Nη2	3.0
Arg153	Asp147	Arg153 Nε:Asp147 Oδ1	3.2
Arg153	Asp147	Arg153 Nη2:Asp147 Oδ2	2.8
Lys169	Arg181	Lys169 O:Arg181 Nη1	3.1
Lys169	Arg181	Lys169 O:Arg181 Nε	3.2
Lys169	Ser199	Lys169 Nζ:Ser199 O	2.6
Lys169	Glu200	Lys169 Nζ:Glu200 Oε1	3.1
**NKR-P1 – chain D**	**NKR-P1 – chain C_{sym}**	**Hydrogen bonds**	**Distance [Å]**
Primary-secondary mode (NKR-P1: chain D, NKR-P1: chain C_{sym})			
Gln100	Ser161	Gln100 N:Ser161 O	2.9
Gln99	Glu162	Gln99 Nε2:Glu162 O	3.0
Asn143	Asn174	Asn143 Nδ2:Asn174 O	3.3
Asn143	Asn174	Asn143 Nδ2:Asn174 Oδ1	2.8
Asn143	Asn176	Asn 143 O:Asn 176 Nδ2	3.4
Ile145	Asn176	Ile 145 O:Asn 176 Nδ2	3.0
Arg146	Ile180	Arg146 Nη1:Ile 180 O	2.8

Supplementary Table 2. Interaction interface residues conserved among homologous NK cell CTL receptor:ligand complexes. Pairs of interacting amino acid residues conserved in the primary interaction mode of human NKR-P1:LLT1, murine NKR-P1B:Clrb, and human NKp65:KACL complexes' crystal structures are listed, together with their mutual distances.

**NKR-P1:LLT1**	**Distance [Å]**	**NKR-P1B:Clrb**	**Distance [Å]**	**NKp65:KACL**	**Distance [Å]**
D/Ser199 O: B/Tyr177 OH	2.6	U/Ser199 O: I/Tyr183 OH	3.0	*Phe160 in place of Tyr*	-
D/Glu205 O: B/Tyr165 OH	3.6	U/Asp205 O: I/Tyr171 OH	2.6	*Phe148 in place of Tyr*	-
D/Asp183 Oδ2: B/Arg175 Nη1	2.9	U/Ser188 Oγ: I/Arg181 Nη1	3.3	B/Ser171 Oγ: A/Arg158 Nη1	2.5
D/Asp183 Oδ2: B/Arg175 Nη2	3.2	U/Ser188 Oγ: I/Arg181 Nη2	3.1	B/Ser171 Oγ: A/Arg158 Nη2	3.1
-	-	U/Ser199 Oγ: I/Arg181 Nη1	3.6	B/Ser182 Oγ: A/Arg158 Nη2	3.4
D/Ser199 O: to Lys169 Nζ in secondary mode	*2.6*	U/Ser199 O: I/Arg181 Nη1	3.4	B/Ser182 O: A/Arg158 Nη2	3.1

Supplementary Table 3. Comparison of the previously proposed NKR-P1:LLT1 binding model with the crystal structure. The table lists point mutations of LLT1 and NKR-P1 residues that had either a detrimental or moderate negative effect on the binding of its partner as determined by SPR analyses in the previous works of Kamishikiryo *et al.* and Kita *et al.*[1,2]. The amino acid interaction pairs proposed in these studies are listed at the bottom. The presence or absence of such residue or interaction pair within the primary or secondary binding mode interface in the herein reported NKR-P1:LLT1 complex crystal structure is indicated with upper indices (P – primary, S – secondary, - – absence).

LLT1	NKR-P1
Detrimental effect on binding	
Lys169Glu $^{P/S}$	Glu162Arg $^{-/-}$
Arg175Glu $^{P/-}$	Asp183Arg $^{P/S}$
Arg180Glu $^{P/-}$	Tyr198Ala $^{P/S}$
Lys181Glu $^{P/-}$	Tyr201Ala $^{P/S}$
	Glu205Arg $^{P/-}$
Moderate effect on binding	
Tyr165Ala $^{P/S}$	Arg181Glu $^{P/S}$
Asn167Ala $^{P/-}$	Glu186Arg $^{-/-}$
Proposed LLT1:NKR-P1 interaction pairs	
Lys169:Glu205 $^{-/-}$	
Arg175:Glu200 $^{P/-}$	
Glu179:Ser193/Thr195 $^{P/-}$	
Tyr177:Tyr198 $^{P/-}$	
Tyr165:Phe152 $^{-/-}$	

Supplementary References

1. Kamishikiryo, J., Fukuhara, H., Okabe, Y., Kuroki, K. & Maenaka, K. Molecular basis for LLT1 protein recognition by human CD161 protein (NKRP1A/KLRB1). *J. Biol. Chem.* **286**, 23823-23830 (2011).
2. Kita, S. et al. Crystal structure of extracellular domain of human lectin-like transcript 1 (LLT1), the ligand for natural killer receptor-P1A. *Eur. J. Immunol.* **45**, 1605-1613 (2015).
