# Supplementary material for: Structure of the human NK cell NKR-P1:LLT1 receptor:ligand complex reveals clustering in the immune synapse
Source: Nat Commun. 2022 Aug 26;13:5022. doi: 10.1038/s41467-022-32577-6 (PMC9418145; doi:10.1038/s41467-022-32577-6)
Supplement: Supplementary file 3 — Description of Additional Supplementary Files [file 41467_2022_32577_MOESM3_ESM.pdf]

Description of Additional Supplementary Files

Supplementary Data 1. OLIGOMER analysis of the individual SAXS curves. Data are divided into two spreadsheets according to the two main resolved peaks obtained in the SEC-SAXS experiment (frames 340-419 and 421-521). The third spreadsheet describes the analysis of the six selected merged data intervals (related to Supplementary Fig. 6). Library of NKR-P1 and LLT1 structural models representing monomers, dimers, and their interacting multimers in various permutations of primary and secondary interaction modes, generated in PyMOL from the herein solved crystal structures by applying symmetry operations, was used for OLIGOMER analysis of the individual scattering curves. The composition and length of structural models are denoted as follows: LLT1 monomer (L), NKR-P1 monomer interacting with LLT1 in primary mode (P), NKR-P1 monomer interacting with LLT1 in secondary mode (S), and NKR-P1 monomer not interacting with LLT1 (N). The OLIGOMER-derived contributing fraction of the utilized model(s) is shown for each scattering curve, together with χ^2 of the fitted superposed curve and predicted Rg value.
