# Supplementary material for: Structure of the human NK cell NKR-P1:LLT1 receptor:ligand complex reveals clustering in the immune synapse
Source: Nat Commun. 2022 Aug 26;13:5022. doi: 10.1038/s41467-022-32577-6 (PMC9418145; doi:10.1038/s41467-022-32577-6)
Supplement: Supplementary file 5 — Reporting Summary [file 41467_2022_32577_MOESM5_ESM.pdf]

nature portfolio

Corresponding author(s): Ondřej Vaněk

Last updated by author(s): Jun 13, 2022

Reporting Summary

Nature Portfolio wishes to improve the reproducibility of the work that we publish. This form provides structure for consistency and transparency in reporting. For further information on Nature Portfolio policies, see our Editorial Policies and the Editorial Policy Checklist.

Statistics

For all statistical analyses, confirm that the following items are present in the figure legend, table legend, main text, or Methods section.

n/a	Confirmed	
☐	☒	The exact sample size (*n*) for each experimental group/condition, given as a discrete number and unit of measurement
☐	☒	A statement on whether measurements were taken from distinct samples or whether the same sample was measured repeatedly
☐	☒	The statistical test(s) used AND whether they are one- or two-sided<br>*Only common tests should be described solely by name; describe more complex techniques in the Methods section.*
☒	☐	A description of all covariates tested
☐	☒	A description of any assumptions or corrections, such as tests of normality and adjustment for multiple comparisons
☐	☒	A full description of the statistical parameters including central tendency (e.g. means) or other basic estimates (e.g. regression coefficient) AND variation (e.g. standard deviation) or associated estimates of uncertainty (e.g. confidence intervals)
☐	☒	For null hypothesis testing, the test statistic (e.g. *F*, *t*, *r*) with confidence intervals, effect sizes, degrees of freedom and *P* value noted<br>*Give P values as exact values whenever suitable.*
☒	☐	For Bayesian analysis, information on the choice of priors and Markov chain Monte Carlo settings
☒	☐	For hierarchical and complex designs, identification of the appropriate level for tests and full reporting of outcomes
☒	☐	Estimates of effect sizes (e.g. Cohen's *d*, Pearson's *r*), indicating how they were calculated

Our web collection on statistics for biologists contains articles on many of the points above.

Software and code

Policy information about availability of computer code

Data collection	X-ray diffraction data were collected at Diamond Light Source (Didcot, UK) beamlines I03 and 21.<br>dSTORM microscopy data were acquired with ZEN (Black edition) v.2.0 software, FACS data were acquired with BD FACSDiva v8.0 software. CD, AUC, and MST data were acquired using the software provided by the instruments' manufacturers, i.e., Applied Photophysics (Pro-Data Chirascan v4.1), Beckman Coulter (ProteomeLab v6.2), and NanoTemper (MO.Control v1.5.3), respectively.
Data analysis	X-ray diffraction data were integrated with XDS software (XDS.INP 0.52, INTEGRATE November 3, 2014) and merged and scaled using Aimless (under CCP4 v6.5.017 software package). Phase problem was solved using BALBES (v1.0.0.Nov_16_2011), Molrep (under CCP4 v6.5.017) and PHASER (under CCP4 v6.5.017). Structures were refined using Refmac5 (v5.8.0131 under CCP4 v6.5.017), with manual editing in Coot v0.8.9. SEC-SAXS data were reduced in Scatter3 v3.1r (available from https://www.bioisis.net/tutorials/9), and the final scattering data were further analyzed using the tools available from the ATSAS suite v2.8.2 software package, including Oligomer (v7.1 under ATSAS 2.8.2). The CCP4 software package is available from https://www.ccp4.ac.uk, and the ATSAS suite is available from https://www.embl-hamburg.de/biosaxs/atsas-online/download.php. Models of protein structures were rendered in PyMOL (v1.8.2.0). Multiple sequence alignment was performed in Clustal Omega (available at https://www.ebi.ac.uk/Tools/msa/clustalo/), and the corresponding graphics was prepared in ESPript 3.0 (available at https://espript.ibcp.fr/ESPript/cgi-bin/ESPript.cgi). CD spectroscopy data were analyzed using the CDNN 2.1 program provided with the Chirascan Plus CD spectropolarimeter (available from http://gerald-boehm.de/download/cdnn). AUC data were analyzed using Sednterp v3.0.3 (available from www.jphilo.mailway.com), Sedfit v16.36 and Sedphat v15.2c software, figures were prepared in GUSSI v1.4.2. MST data were analyzed in PALMIST v1.5.8, Sedphat v15.2c, and GUSSI v1.4.2. Sedfit and Sedphat are available from https://sedfitsedphat.nibib.nih.gov/software/default.aspx, while GUSSI and PALMIST are available from https://www.utsouthwestern.edu/labs/mbr/software. dSTORM data were processed using ImajeJ (Fiji) v.1.52k with ThunderSTORM plug-in v.1.3 (available from https://zitmen.github.io/thunderstorm), cluster analysis was performed in ClusterViSu v.1.1.2 (available from https://github.com/andronovl/SharpViSu), statistical analysis was performed in OriginPro 2018 (v.b9.5.1.195). FACS data were evaluated using FlowJo v.10.6 software.

For manuscripts utilizing custom algorithms or software that are central to the research but not yet described in published literature, software must be made available to editors and reviewers. We strongly encourage code deposition in a community repository (e.g. GitHub). See the Nature Portfolio guidelines for submitting code & software for further information.

Data

Policy information about availability of data

All manuscripts must include a data availability statement. This statement should provide the following information, where applicable:

- Accession codes, unique identifiers, or web links for publicly available datasets
- A description of any restrictions on data availability
- For clinical datasets or third party data, please ensure that the statement adheres to our policy

The refined coordinate and structure factor files for the X-ray crystal structures reported in this study have been validated by the Protein Data Bank (www.wwpdb.org) and deposited there under the accession numbers of 5MGR (NKR-P1_glyco; doi: 10.2210/pdb5MGR/pdb), 5MGS (NKR-P1_deglyco; doi: 10.2210/pdb5MGS/pdb), and 5MGT (NKR-P1:LLT1; doi: 10.2210/pdb5MGT/pdb). Links to the other PDB entries in this paper are: 2BPD (doi: 10.2210/pdb2BPD/pdb), 2CL8 (doi: 10.2210/pdb2CL8/pdb), 3FF7 (doi: 10.2210/pdb3FF7/pdb), 3T3A (doi: 10.2210/pdb3T3A/pdb), 4IOP (doi: 10.2210/pdb4IOP/pdb), 4QKI (doi: 10.2210/pdb4QKI/pdb), 5J2S (doi: 10.2210/pdb5J2S/pdb), and 6E7D (doi: 10.2210/pdb6E7D/pdb). Diffraction data have been deposited in the SBGrid Data Bank under the codes 778 (NKR-P1_glyco; doi:10.15785/SBGRID/778); 779 (NKR-P1_deglyco, doi:10.15785/SBGRID/779) and 780 (NKR-P1:LLT1; doi:10.15785/SBGRID/780). Diffraction data from the SEC-SAXS experiment have been deposited to Mendeley Data (doi:10.17632/268ww2m4j3.1). The total output of the OLIGOMER analysis of the SEC-SAXS data is available as Supplementary Data 1. Source data are provided with this paper.

Field-specific reporting

Please select the one below that is the best fit for your research. If you are not sure, read the appropriate sections before making your selection.

☒ Life sciences ☐ Behavioural & social sciences ☐ Ecological, evolutionary & environmental sciences

For a reference copy of the document with all sections, see nature.com/documents/nr-reporting-summary-flat.pdf

Life sciences study design

All studies must disclose on these points even when the disclosure is negative.

Sample size	No statistical methods were used to predetermine sample size. In the dSTORM experiment, the sample size reflects datasets commonly used in this type of experiments [Oszmiana et al., Cell Rep 2016;15(9):1957-72; doi: 10.1016/j.celrep.2016.04.075] – i.e., ca more than 20 cells per each condition. Our data represent 46 NKR-P1+ control cells and 41 or 47 NKR-P1+ cells incubated with LLT1 or LLT1(SIM). The cytotoxicity assay experiment was repeated three times (three independent donors) as it is common for such biological experiments [Peipp et al., Scand J Immunol 2017;86(4):196-206; doi: 10.1111/sji.12581]. X-ray diffraction data were collected on several single crystals, sample size was determined according to the beamtime availability and with regards to the established statistical values describing the obtained data from these crystals. SEC-SAXS diffraction data were collected on single SEC run of in-solution samples. Sample size was determined according to the beamtime availability and quality of the resulting diffraction data. Sample size for AUC and MST measurements was determined based on the estimated Kd value and estimated concentration range that would be necessary to obtain complete titration binding isotherm.
Data exclusions	No data were excluded on purpose. The only excluded data (dSTROM, FACS) were the data with low quality or when an error occured during data acquisition.
Replication	dSTORM experiment was performed in at least four independent runs (in each run, data were acquired for NKR-P1+ control cells and experimental condition, to assure consistent data acquisition quality). For FACS data acquisition, technical triplicate (or duplicate) was prepared in each of the three independent experiments. MST measurements were performed in triplicate (three independent experiments). Successful crystallization experiments were performed more than ten times for proteins with different glycosylation profiles. CD spectroscopy measurement, AUC sedimentation analyses, and SEC-SAXS data collection were performed only once. All attempts at replication were successful.
Randomization	For structure refinement, a subset of test reflections was selected at random for cross validation. Otherwise, sample randomization was not applicable; however, the data from different experimental conditions were always treated the same way.
Blinding	Blinding was not applicable; however, the data from different experimental conditions were always treated the same way.

Reporting for specific materials, systems and methods

We require information from authors about some types of materials, experimental systems and methods used in many studies. Here, indicate whether each material, system or method listed is relevant to your study. If you are not sure if a list item applies to your research, read the appropriate section before selecting a response.

Materials & experimental systems

n/a	Involved in the study	
☐	☒	Antibodies
☐	☒	Eukaryotic cell lines
☒	☐	Palaeontology and archaeology
☒	☐	Animals and other organisms
☐	☒	Human research participants
☒	☐	Clinical data
☒	☐	Dual use research of concern

Methods

n/a	Involved in the study	
☒	☐	ChIP-seq
☐	☒	Flow cytometry
☒	☐	MRI-based neuroimaging

Antibodies

Antibodies used: Alexa Fluor647 labeled anti-human CD161 antibody (clone HP-3G10; BioLegend, cat. no. 339910, lot no. B282422). Final concentration was 10 µg/ml, which means 20x diluted Ab stock solution).

Validation: The antibody was verified by the supplier. Validation data are available on the manufacturer's website (https://www.biolegend.com/en-us/products/alexa-fluor-647-anti-human-cd161-antibody-5741).

Eukaryotic cell lines

Policy information about cell lines

Cell line source(s): HEK293S GnTI(–) cell line was purchased from ATCC (CRL3022). K562 cells were kindly provided by Institute of Molecular Genetics of the Czech Academy of Sciences, Prague.

Authentication: No cell authentication was used.

Mycoplasma contamination: All cell lines tested negative for mycoplasma.

Commonly misidentified lines (See ICLAC register): No misidentified cell lines were used.

Human research participants

Policy information about studies involving human research participants

Population characteristics: The donors of the blood (buffy coats for NK cell isolation) were anonymous.

Recruitment: Buffy coats for human NK cell isolation were purchased from the Institute of Hematology and Blood Transfusion (IHBT, Prague, Czech Republic) as material for research use based on the bilateral cooperation agreement between IHBT and Charles University. The IHBT arranged the consent of the donors.

Ethics oversight: The Institute of Hematology and Blood Transfusion (Prague, Czech republic) arranged the consent of the donors according to their internal guidelines and ethic regulations.

Note that full information on the approval of the study protocol must also be provided in the manuscript.

Flow Cytometry

Plots

Confirm that:

☒ The axis labels state the marker and fluorochrome used (e.g. CD4-FITC).

☒ The axis scales are clearly visible. Include numbers along axes only for bottom left plot of group (a 'group' is an analysis of identical markers).

☒ All plots are contour plots with outliers or pseudocolor plots.

☒ A numerical value for number of cells or percentage (with statistics) is provided.

Methodology

Sample preparation: Primary NK cells were isolated by negative selection using an NK cell isolation kit (Miltenyi Biotec) and cultivated overnight in RPMI1640 (supplemented with 10% FCS, 100 units/ml penicillin, 100 µg/ml streptomycin, and 80 ng/ml IL-2). For the cytotoxicity assay, NK cells were incubated with K562 cells (stained with CellTrace Violet, CTV, Proliferation Kit, Thermo Fisher) in presence or absence of LLT1 or LLTI(SIM) protein for 4 hours. Cells were then washed and stained with 7-AAD, as a cell viability reagent.

Instrument	BD LSR II flow cytometer (BD Biosciences)
Software	FACS data were acquired with BD FACSDiva Software (v.8.0) and evaluated using FlowJo software (v.10.6.).
Cell population abundance	Samples contained 400.000 NK cells and 10.000 K562 cells (ratio 40:1 efector vs. target cells). The result (percentage of living cells) was derived from the population of CTV+ cells (K562 cells were pre-stained with proliferation reagent), an average number of CTV+ was 3700, ranging from 1000 - 7500 cells (the very exceptional minimum was 800).
Gating strategy	First, we identified a population of cells from all events, then we identified subset of single cells. Out of the single cells, we gated to CTV+ cells (K562 cells). Finally, CTV+ were divided into 7-AAD positive (dead) and negative (live) cells.

☒ Tick this box to confirm that a figure exemplifying the gating strategy is provided in the Supplementary Information.
